# Vegetation impact on atmospheric moisture transport under increasing land-ocean temperature contrasts

**DOI:** 10.1016/j.heliyon.2022.e11173

**Published:** 2022-10-20

**Authors:** Anastassia M. Makarieva, Andrei V. Nefiodov, Antonio Donato Nobre, Douglas Sheil, Paulo Nobre, Jan Pokorný, Petra Hesslerová, Bai-Lian Li

**Affiliations:** a Theoretical Physics Division, Petersburg Nuclear Physics Institute, Gatchina, St. Petersburg, 188300, Russia; b Institute for Advanced Study, Technical University of Munich, Lichtenbergstrasse 2 a, Garching, D-85748, Germany; c USDA-China MOST Joint Research Center for AgroEcology and Sustainability, University of California, Riverside, CA 92521-0124, USA; d Centro de Ciência do Sistema Terrestre INPE, São José dos Campos, São Paulo, 12227-010, Brazil; e Forest Ecology and Forest Management Group, Wageningen University & Research, PO Box 47, Wageningen, 6700 AA, the Netherlands; f Center for International Forestry Research (CIFOR), Kota Bogor, 16115, Jawa Barat, Indonesia; g Faculty of Environmental Sciences and Natural Resource Management, Norwegian University of Life Sciences, Ås, Norway; h Center for Weather Forecast and Climate Studies INPE, São José dos Campos, São Paulo, 12227-010, Brazil; i ENKI, o.p.s., Dukelská 145, Třeboň, 379 01, Czech Republic

**Keywords:** Drought, Heatwaves, Vegetation cover, Evapotranspiration, Wind power

## Abstract

Destabilization of the water cycle threatens human lives and livelihoods. Meanwhile our understanding of whether and how changes in vegetation cover could trigger transitions in moisture availability remains incomplete. This challenge calls for better evidence as well as for the theoretical concepts to describe it. Here we briefly summarize the theoretical questions surrounding the role of vegetation cover in the dynamics of a moist atmosphere. We discuss the previously unrecognized sensitivity of local wind power to condensation rate as revealed by our analysis of the continuity equation for a gas mixture. Using the framework of condensation-induced atmospheric dynamics, we then show that with the temperature contrast between land and ocean increasing up to a critical threshold, ocean-to-land moisture transport reaches a tipping point where it can stop or even reverse. Land-ocean temperature contrasts are affected by both global and regional processes, in particular, by the surface fluxes of sensible and latent heat that are strongly influenced by vegetation. Our results clarify how a disturbance of natural vegetation cover, e.g., by deforestation, can disrupt large-scale atmospheric circulation and moisture transport: an increase of sensible heat flux upon deforestation raises land surface temperature and this can elevate the temperature difference between land and ocean beyond the threshold. In view of the increasing pressure on natural ecosystems, successful strategies of mitigating climate change require taking into account the impact of vegetation on moist atmospheric dynamics. Our analysis provides a theoretical framework to assess this impact. The available data for the Northern Hemisphere indicate that the observed climatological land-ocean temperature contrasts are close to the threshold. This can explain the increasing fluctuations in the continental water cycle including droughts and floods and signifies a yet greater potential importance for large-scale forest conservation.

## Highlights

• Consideration of condensation dynamics reveals temperature-related tipping points.
• Additional heat over land can block oceanic moisture import causing severe drought.
• As the land warms faster than the ocean, these tipping thresholds approach.
• Deforestation increases sensible heat and exacerbates these water cycle extremes.

## Graphical abstract

**Figure:**
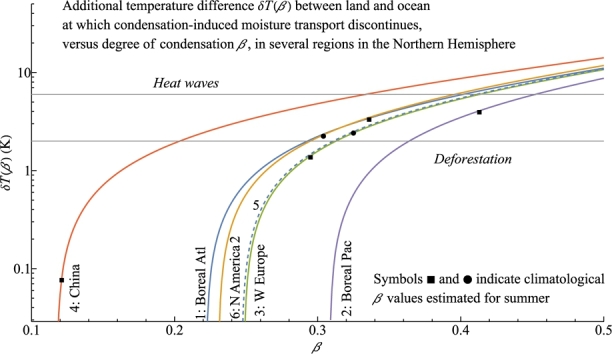

## 1 Introduction

Reliable water is crucial for human life. Long-term data indicate that in recent decades many regions of the world, including Eurasia and Western Europe, have been steadily losing soil moisture during the vegetative season (Gu et al., 2019). Other regions have experienced unprecedented droughts (Marengo and Espinoza, 2016) and floods (Cornwall, 2021).

Drought stresses plants and modifies their influence on the water cycle and climate. These water-vegetation feedbacks present both risks and opportunities. The risks include the potential for a complete switch from a wet to an arid climate state. The opportunities arise in the ability to slow down and reverse aridification.

Given the scale and nature of these trends and opportunities, our understanding of the underlying feedbacks and mechanisms remains inadequate. Here we will consider the concepts of *biotic pump* (Makarieva and Gorshkov, 2007) and the associated condensation-induced atmospheric dynamics (CIAD). These concepts were invoked to explain spatial and temporal precipitation patterns in various regions (e.g., Andrich and Imberger, 2013; Poveda et al., 2014; Molina et al., 2019). These triggered multiple discussions (Meesters et al., 2009; Makarieva and Gorshkov, 2009; Angelini et al., 2011; Makarieva et al., 2013a; Jaramillo et al., 2018, Jaramillo et al., 2019; Makarieva et al., 2019; Pearce, 2020). The main implication of the biotic pump for the vegetation-atmosphere dynamics is that large-scale forests, by generating and maintaining atmospheric moisture through transpiration, can power the ocean-to-land winds and the associated atmospheric moisture transport. Essentially, water vapor removal from the gas phase produces non-equilibrium pressure gradients that generate both vertical and horizontal wind. Using the CIAD framework, we will explore how atmospheric moisture transport can be affected by the changing land-ocean temperature contrasts.

In this temperature-related context, theoretical studies of vegetation-water relations have long featured conceptual controversies. Charney (1975) proposed that increased albedo from vegetation dieback should cool land, reduce the land-ocean temperature gradient, weaken ocean-to-land moisture advection and thus further enhance droughts. Ripley (1976) objected that drying warms the land surface via a reduction in evapotranspiration (latent heat flux): a negative rather than a positive feedback. Charney (1976) replied that extra cooling over the drier land will manifest itself in the upper atmosphere and not on the surface, but agreed that the ultimate land-ocean temperature contrasts are model-dependent – as was illustrated by later studies (Claussen, 1997).

**Figure 1:**
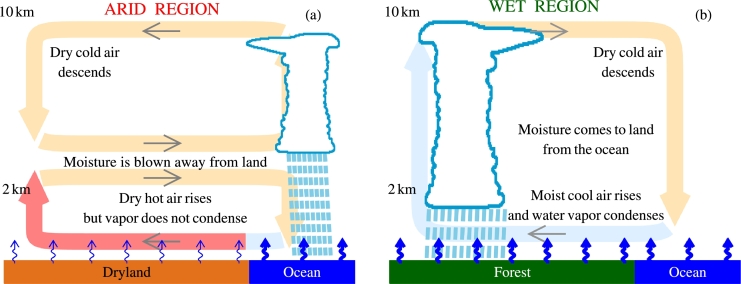
Air circulation between (a) the ocean and a hot and dry land (cf. Fig. 3 of Charney, 1975) and (b) the ocean and a cool and moist land. The ocean and the forest have higher evaporation rates (thick blue arrows) than the dryland (thin blue arrows). The vertical profile of the pressure differences between the atmosphere over land and over the ocean in our (a) and (b) can be qualitatively illustrated by, respectively, Fig. 2c and Fig. 2b of Makarieva et al. (2015). In (a), there is a pressure surplus over land in the lower troposphere at the height of the outflow, and pressure shortages above and below; in (b), there is a pressure shortage in the lower atmosphere and a pressure surplus in the upper atmosphere.

This debate about evapotranspiration, latent heat and related temperature differences persisted through many years. Discussions focused on whether and how changes in vegetation could trigger an abrupt switch of ocean-to-land air circulation (Fig. 1). Levermann et al. (2009) proposed that monsoonal regimes can switch via a tipping point involving a positive feedback of moisture advection on the land-ocean temperature contrast. The idea was that the more moisture comes from the colder ocean to condense over the warmer land, the more latent heat is released warming land even further. Boos and Storelvmo, 2016a, Boos and Storelvmo, 2016b used a global climate model to demonstrate that such a scenario is physically implausible. To descend over the ocean, the air warmed by latent heat release over land must give the extra heat away (Fig. 1b) – otherwise the circulation would stop. The finite cooling rate limits the enhancement in circulation by latent heat release. In their reply, Levermann et al. (2016) did not specify any mechanism that could enable warm air to overcome buoyancy and descend. The controversy persists: recently, Boers et al. (2017) proposed a similar drought-related tipping point but neglected previous discussions (Levermann et al., 2009, Levermann et al., 2016; Boos and Storelvmo, 2016a, Boos and Storelvmo, 2016b).

In a related context, Kuo et al. (2017) investigated the causality between deep convection and high tropospheric moisture content. Is high water vapor content a consequence of the atmosphere being moistened by convection, or, conversely, does high water vapor content trigger convection? Kuo et al.'s (2017) modelling results appeared to support the latter. Similarly, on a much wider spatial scale, another study concluded that high transpiration by the Amazon rainforest during the late dry season moistens the atmosphere and triggers the beginning of the Amazon wet season well in advance of the arrival of the Intertropical Convergence Zone (Wright et al., 2017). Thus, the switch between circulation patterns in Fig. 1 can be enforced by atmospheric moistening via evapotranspiration. Pradhan et al. (2019) also found that the onset of summer monsoon in Northeast India is preceded by enhanced transpiration. Recent studies indicate that evaporation during rainfall can be significant, highlighting the tight coupling between these processes (Murakami, 2021; Jiménez-Rodríguez et al., 2021).

One long-standing challenge in the analyses of vegetation-atmosphere feedbacks has been the inadequate representation of continental moisture convergence in global models (Hagemann et al., 2011). In the steady state, the net amount of atmospheric moisture brought to land by winds from the ocean (moisture convergence) must match the runoff from land to the ocean. While runoff R is measured directly, moisture convergence C is model-derived. The match tends to be imperfect: instead of the equality, R=C, implied by mass conservation, the discrepancy between C and R can be of the order of 100% (as it is, for example, for the Amazon basin (Hagemann et al., 2011)). For the continental moisture budget, C=P−E, the underestimate of moisture convergence C implies that either precipitation P is underestimated, or evapotranspiration E is overestimated, or both. Evapotranspiration is generally the least certain component of the terrestrial water cycle (e.g., Lugato et al., 2013). Reliable analyses of vegetation-atmosphere feedbacks and their spatial and temporal propagation in large river basins such as the Amazon requires these inconsistencies to be resolved (e.g., Salati and Nobre, 1991; Zemp et al., 2017a, Zemp et al., 2017b; Molina et al., 2019; Ruiz-Vásquez et al., 2020).

Incomplete understanding of vegetation-water feedbacks as related to temperature have implications for models and resulting global climate projections. Recent studies demonstrate that the warming that results from reduced transpiration more than compensates for the cooling that results from increased albedo, such that deforestation results in an elevation of local surface temperatures during the vegetative season by up to several kelvin (Huryna and Pokorný, 2016; Alkama and Cescatti, 2016; Hesslerová et al., 2018). The net change of global mean surface temperature resulting from changes of albedo and transpiration following a large-scale deforestation is estimated at about ±0.05 K (the sign varies among models) (Winckler et al., 2019). The gross changes, however, are more than an order of magnitude larger and comparable in magnitude to observed global warming. The nature of this fine balance between physically distinct effects has not been explained and requires an investigation. If it turns out to have resulted from model tuning, the impact of deforestation on climate destabilization may be greatly underestimated. The first systematic analyses of the forest control of cloud cover indicate that the previous assessments of the forest contribution to the maintenance of global surface temperature require a re-evaluation (Duveiller et al., 2021; Cerasoli et al., 2021).

The preceding brief account demonstrates the many challenges and unresolved problems surrounding the field of moist atmospheric dynamics. The recent trajectory of environmental and climate research revolved more around the development of numerical models and empirical data gathering. Considering achievements to date, leading researchers have begun to re-emphasize the need for strong theoretical knowledge as a framing and foundation for effective climate science (Emanuel, 2020). Theory is required to judge and understand the adequacy and outputs of numerical models. Vegetation-atmosphere feedbacks, with their complexity and profound implications for the humanity's well-being, appear to be the topic where new theoretical approaches could be particularly useful.

In Section 2 we briefly introduce the main equations of CIAD with an emphasis on how local wind power can be estimated from the continuity equation. We highlight and explain the sensitivity of the wind power to the formulation of the condensation rate. Next in Section 3 we formulate CIAD in an integral form that allows the estimation of the role of the horizontal temperature differences for the moisture transport. In Sections 4 and 5 we use observation based climatological data to estimate the relevant quantities for several regions in the Northern Hemisphere. Regional cooling provided by the transpiring vegetation cover can buffer the land-ocean temperature differences and prevent the drought-related tipping points. Conversely, deforestation and the associated extra warming can trigger such extremes of the atmospheric moisture transport. In the concluding sections we outline a few implications of the obtained results for current climate policies.

## 2 Condensation rate and wind power

Two vertical scales characterize our atmosphere. One is the hydrostatic height h≡−p/(∂p/∂z)=RT/Mg∼10 km determined by the interplay between the gravitational and internal energy of the atmospheric gases. Another is the vertical scale height for the condensable gas, water vapor, $h_{c}\equiv -p_{v}/(\partial p_{v}/\partial z)=RT^{2}/L\Gamma ∼2$ km, that is determined by the interplay between the cooling rate of ascending air and latent heat release during any resulting condensation. Here *p* is air pressure, $p_{v}$ is partial pressure of saturated water vapor, R=8.3 J/mol/K is the universal gas constant, *T* is absolute temperature, M≃29 g/mol is mean molar mass of atmospheric gases, *g* is the acceleration of gravity, L≃45 kJ/mol is the latent heat of vaporization, Γ≡−∂T/∂z≃6.5 K/km is the vertical lapse rate of air temperature.

That $h_{c}\ll h$ means that the vertical gradient of water vapor partial pressure is strongly non-equilibrium. This allows the formulation of the rate of potential energy release (${\text{W/m}}^{3}$) during condensation in the ascending air as

(1) $$s\equiv -wp_{v}\left(\frac{1}{h_{c}}-\frac{1}{h}\right)\equiv wp\frac{\partial \gamma }{\partial z}\equiv -wf_{e},\;f_{e}\equiv -\frac{\partial p_{v}}{\partial z}+\frac{p_{v}}{p}\frac{\partial p}{\partial z}\equiv \frac{p_{v}}{h_{\gamma }},$$

where $h_{\gamma }\equiv -\gamma /(\partial \gamma /\partial z)={\left(h_{c}^{-1}-h^{-1}\right)}^{-1}$, $\gamma \equiv p_{v}/p$, *w* is the vertical air velocity and $f_{e}$ has the meaning of a vertical force associated with the non-equilibrium partial pressure gradient of water vapor.

The main proposition of the biotic pump concept – and the underlying condensation-induced atmospheric dynamics – is a power source for atmospheric circulation (Makarieva and Gorshkov, 2007, Makarieva and Gorshkov, 2010; Makarieva et al., 2019). Applied locally in a hydrostatic horizontally isothermal saturated atmosphere, where all wind power is generated by horizontal pressure gradients, this proposition takes the form

(2) $$u\frac{\partial p}{\partial x}=s,$$

where *u* is horizontal air velocity directed along *x*-axis.

Theoretical relation (2) agreed with observations in different atmospheric contexts, including general atmospheric circulation, the Amazon basin and the more compact circulation patterns like hurricanes and tornadoes (see Makarieva et al. (2019) and references therein). In these compact vortices partial pressure $p_{v}$ of water vapor sets the scale for maximum wind velocity $u_{\max}=\sqrt{2p_{v}/\rho }∼70$ m/s, where $\rho ≃1\;{\text{kg/m}}^{3}$ is air density. This generality – i.e., the validity of Eq. (2) across several orders of magnitude for vertical velocity *w* – is satisfying for a theorist and incentivizes efforts to understand the underlying mechanisms and their implications more comprehensively.

Noting the different equivalent expressions for *s* (1), we observe similarity between *s* and the term containing vertical velocity in the continuity equation expressed in terms of pressure:

(3) $$w\left(\frac{\partial p_{v}}{\partial z}-\frac{p_{v}}{p_{d}}\frac{\partial p_{d}}{\partial z}\right)+u\left(\frac{\partial p_{v}}{\partial x}-\frac{p_{v}}{p_{d}}\frac{\partial p_{d}}{\partial x}\right)=\sigma .$$

Here $p_{d}=p-p_{v}$ is the partial pressure of dry air, σ=SRT is the rate of phase transitions in power units (${\text{W/m}}^{3}$), S (${\text{mol/m}}^{3}\text{/s}$) is the molar rate of phase transitions (see Eqs. (1), (6) and (8) of Gorshkov et al. (2012) and Eq. (A.4) in Appendix A). Such a representation of the continuity equation is only possible for an ideal gas with its equation of state relating molar density and pressure.

The relation between *s* (1) and

(4) $$s_{d}\equiv -wp_{v}\left(\frac{1}{h_{c}}-\frac{1}{h_{d}}\right)\equiv w\left(\frac{\partial p_{v}}{\partial z}-\frac{p_{v}}{p_{d}}\frac{\partial p_{d}}{\partial z}\right)\equiv wp_{d}\frac{\partial \gamma_{d}}{\partial z},$$

where $\gamma_{d}\equiv p_{v}/p_{d}$, $h_{d}\equiv RT/M_{d}g$, $M_{d}$ is the molar mass of dry air, is

(5) $$s\equiv (1-\gamma )s_{d}.$$

They differ by a small magnitude $\gamma \equiv p_{v}/p\ll 1$.

At constant relative humidity, $p_{v}$ grows with increasing temperature *T* in accordance with the Clausius-Clapeyron equation

(6) $$\frac{dp_{v}}{p_{v}}=\xi \frac{dT}{T},\;\xi \equiv \frac{L}{RT}.$$

Assuming relative humidity to be constant and the air to be isothermal in the horizontal plane,^1^ such that $\partial p_{v}/\partial x=0$, we can write the continuity equation (3) in the following form:

(7) $$u\frac{\partial p}{\partial x}=u\frac{\partial p_{d}}{\partial x}=\frac{1}{\gamma_{d}}\left(s_{d}-\sigma \right).$$

Since the vertical motions associated with adiabatic cooling are the most important mechanisms that bring moist air to saturation, one can argue that condensation rate can be approximated as

(8) $$\sigma \equiv \alpha s_{d},$$

where α≲1 (see, e.g., Jaramillo et al., 2019). Then Eq. (7) can be re-written as

(9) $$u\frac{\partial p}{\partial x}=\frac{s_{d}}{\gamma_{d}}(1-\alpha ),$$

where $\gamma_{d}\equiv p_{v}/p_{d}$. Equation (9) contains a product of a large factor $\gamma_{d}^{-1}∼10^{2}$ and an unknown small factor 1−α≪1. Thus, assuming that a certain (a priori unknown) α≃1 matches the observations, a mere 10% reduction of *α*, while still obeying α≃1, would lead to an order of magnitude overestimate of u∂p/∂x. Conversely, any $p_{d}/p<\alpha <1$ will produce unrealistically low values of u∂p/∂x (down to zero).

To illustrate this, putting $\alpha =p_{d}/p+\Delta \alpha$ into Eq. (9) and taking into account Eq. (5), we obtain

(10) $$u\frac{\partial p}{\partial x}=\frac{s_{d}}{\gamma_{d}}\left(1-\frac{p_{d}}{p}-\Delta \alpha \right)=s\left(1-\frac{p}{p_{v}}\Delta \alpha \right).$$

In the atmosphere of Earth with a typical value of $p_{v}∼20$ hPa, we have $p/p_{v}=50$ and $p_{d}/p=0.98$. With an exemplary Δα=0.018, we have α=0.998. Then the term in braces in the right-hand side of Eq. (10) is equal to 0.1 and we obtain a wind power ten times less than the observed. Conversely, with Δα=−0.2 and α=0.78, the term in braces is equal to 11 and we obtain an order of magnitude higher wind power. In both cases, α=0.998 and α=0.78 the specification α<1 and are sufficiently close to unity to satisfy the stipulation that the vertical motions and gradient make a dominant contribution to condensation rate (8). Nonetheless, the derived wind powers differ greatly — the 20% change in condensation rate *σ* (8) relative to $s_{d}$ has caused wind power to vary by two orders of magnitude. While it follows from Eq. (2), that α≲1 in the continuity equation (10), the main dynamic equation of CIAD, Eq. (2), cannot be derived, even approximately, from the parameterization α≲1 (cf. Jaramillo et al., 2019).

We note that condensation-induced power *s* (1) was introduced from basic principles without referring to the continuity equations (A.1a), (A.1b). Thus, its relation to condensation rate *σ* (A.4) is not *a priori* obvious. Using Eq. (2) to replace u∂p/∂x with *s* in Eq. (7) gives s=σ, such that

(11) $$u\frac{\partial p}{\partial x}=\sigma .$$

While the wind power estimated from the continuity equation is extremely sensitive to the formulation of the *a priori* unknown condensation rate *σ*, realistic values of wind power are consistent with the continuity equation under the assumption that wind power is exactly equal to condensation rate. This is an independent theoretical argument in favor of CIAD.

In the CIAD framework, the high sensitivity of wind power to the magnitude of *σ* can be interpreted as follows. Let us consider the first row in Table 1. If condensation rate is put equal to *s* (1), then it follows from the continuity equation (3) that the wind power generated by the *horizontal* pressure gradient equals condensation rate. The physical justification for the expression for *s* (1) consists in the idea that the gradient of the partial pressure of water vapor is non-equilibrium *relative to the hydrostatic equilibrium of air as a whole*. When the air as a whole is in hydrostatic equilibrium, the wind power generated by the vertical pressure gradient is zero (the upward pressure gradient force is compensated by gravity).

**Table 1:** Physical meaning of the two expressions for condensation rate.

Equilibrium	Condensation rate *σ*	“Horizontal” power	“Vertical” power
$\frac{\partial p}{\partial z}+\rho g=0$	$s\equiv wp\frac{\partial \gamma }{\partial z}$	$u\frac{\partial p}{\partial x}=s$	$w\left(\frac{\partial p}{\partial z}+\rho g\right)=0$
$\frac{\partial p_{d}}{\partial z}+\rho_{d}g=0$	$s_{d}\equiv wp_{d}\frac{\partial \gamma_{d}}{\partial z}$	$u\frac{\partial p}{\partial x}=0$	$w\left(\frac{\partial p}{\partial z}+\rho g\right)=s_{d}+wp_{v}\frac{\epsilon }{h_{d}}$

Here ρ=MN and $\rho_{d}=M_{d}N_{d}$ are the densities of total air and dry air, respectively; $\epsilon \equiv M_{v}/M_{d}-1$. *If* one sets condensation rate as indicated, then the expression for the “horizontal” wind power follows from the continuity equation and $\partial p_{v}/\partial x=0$. Assuming *additionally* the equilibrium condition yields the expression for the “vertical” wind power.

Now let us consider the second row in Table 1. As compared to the first row, in the expression for condensation rate total pressure *p* is replaced by partial pressure $p_{d}$ of dry air, $\sigma =s_{d}$. In this case it follows from the continuity equation that the wind power generated by the horizontal pressure gradient is zero. Indeed, putting α=1 in Eq. (9) (or, equivalently, Δα=γ in Eq. (10)), gives u∂p/∂x=0.

If we assume that now the dry air is in hydrostatic equilibrium (which would justify using $p_{d}$ instead of *p* in the expression for *σ*), we find that now the wind power generated by the *vertical* pressure gradient is equal to condensation rate – plus an additional term proportional to the difference in the molar masses of the water vapor and dry air. This term is relatively small and its physical nature is not related to condensation.^2^ In an atmosphere where $M_{v}=M_{d}=M$ the symmetry would be exact: if the “horizontal” wind power is equal to condensation rate, then the “vertical” one is zero, and vice versa.

As condensation rate changes from *s* to $s_{d}$ (4), the wind power generated by the horizontal pressure gradient diminishes from *s* to zero, while the wind power generated by the vertical pressure gradient grows from zero to, approximately, $s_{d}$. (The atmosphere changes then from a hydrostatic to a non-hydrostatic with the non-equilibrium vertical pressure difference of the order of $p_{v}$.) At intermediate values the power of condensation is allocated to both vertical and horizontal dimensions. These considerations indicate that equations (2) and (11) should remain valid for describing condensation-induced circulation patterns if the kinetic energy of the vertical motion is much less than the kinetic energy of the horizontal motion. For example, it remains valid even in tornadoes where the air is non-hydrostatic, but the squared vertical velocity is still a few times less than the squared horizontal velocity (e.g., Makarieva et al., 2011, Fig. 1B).

## 3 Wind power and horizontal temperature gradient

Applying Eq. (11) to the non-isothermal case, i.e., solving it together with the continuity equation in the form of Eq. (3), leads to the following modification of Eq. (2) (see Makarieva et al. (2014a) and Appendix A for derivation details)

(12) $$u\frac{\partial p}{\partial x}=s+u\frac{\partial p_{v}}{\partial x}=wp\frac{\partial \gamma }{\partial z}+u\frac{\partial p_{v}}{\partial x}.$$

This shows that if the partial pressure of water vapor grows along the horizontal air streamline, the condensation-induced wind power is diminished. While condensation reduces air pressure, evaporation adds gas to the flow and thus increases air pressure along the streamline inhibiting the condensation-induced air flow.

Using the Clausius-Clapeyron equation (6) we can re-write Eq. (12) as follows (Makarieva and Gorshkov, 2010; Makarieva et al., 2014a):

(13) $$-\frac{\partial p}{\partial x}=\frac{w}{u}\frac{p_{v}}{h_{\gamma }}-\frac{\partial p_{v}}{\partial x},\;\frac{\partial p_{v}}{\partial x}=p_{v}\frac{\xi }{T}\frac{\partial T}{\partial x}.$$

Linearizing Eq. (13) by assuming that $w/u∼h_{w}/l$, where $h_{w}$ and *l* are the characteristic vertical and horizontal scales of the moisture inflow in the lower atmosphere, and ∂p/∂x∼Δp/l, $\partial p_{v}/\partial x∼\Delta p_{v}/l$, ∂T/∂x∼ΔT/l, we obtain

(14) $$-\Delta p(\beta )=p_{vA}\frac{h_{w}}{h_{\gamma }}-p_{vD}\xi \frac{\Delta T}{T}=p_{vD}\left[\beta -\xi \frac{\Delta T}{T}(1-\beta )\right],$$

where $\Delta p(\beta )\equiv p_{A}-p_{D}$ and $\Delta p_{v}\equiv p_{vA}-p_{vD}=p_{vD}\xi \Delta T/T$. The quantities of pressure with indices D and A refer to the region that exports moisture (the “donor”) and the region that receives this moisture (the “acceptor”), respectively. Factor $\beta \equiv h_{w}/h_{\gamma }≲1$ corresponds to 1−ζ of Makarieva et al. (2013b) and describes the completeness of condensation in the ascending air, i.e., the share of water vapor that has condensed by the altitude when the air flow changes its horizontal direction (e.g., for the schematic circulation patterns in Figs. 1a and 1b we have, respectively, $h_{w}=2$ km and 10 km).

Equation (14) shows that, for a given β<1, when temperature increases significantly along the horizontal air flow, the negative pressure difference Δp<0 that drives the flow diminishes and, at sufficiently large Δ*T*, can become zero. In this situation, condensation in the ascending air removes as much water vapor as is added to the horizontally moving air near the surface. As a result, the warmer area is locked for condensation-induced air circulation and the condensation-induced moisture inflow ceases. Equation (14) is a manifestation of the general principle that if condensation and evaporation are *not* spatially separated (e.g., if evaporation in the acceptor region is compensated by condensation), no condensation-induced circulation can develop.

When condensation is complete (β=1), all the additional water vapor that evaporates into the air as it moves from the donor to acceptor region ultimately condenses in the acceptor region. The temperature gradient makes no impact on Δ*p*.

Under global climate change, land surface is warming faster than the ocean due to its lower heat capacity and to deforestation that reduces transpiration and elevates surface temperatures during the warmer season (e.g., Alkama and Cescatti, 2016). Thus, the temperature differences between land (that receives moisture from the ocean) and the ocean (which supplies moisture to land) can be expected to grow. It is thus important to estimate observed Δ*T* values to find out whether major ocean-to-land moisture flows may be close to a tipping point (when the term in square brackets in Eq. (14) becomes zero).

## 4 Data and methods

**Figure 2:**
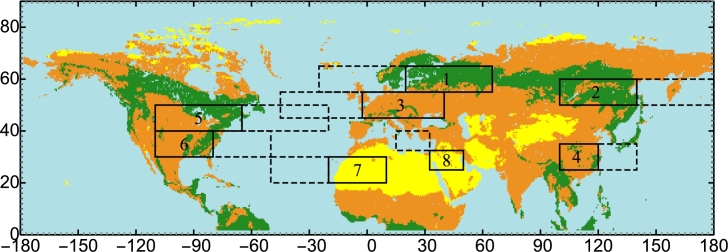
Regions in the Northern Hemisphere where the land-ocean temperature contrasts Δ*T* were investigated: 1: Boreal Atlantic, 2: Boreal Pacific, 3: Western Europe, 4: China, 5: North America 1, 6: North America 2, 7: Sahara, 8: the inner part of the Arabian Peninsula. Different types of vegetation cover (with blue indicated permanent water/ice, yellow – unvegetated or sparsely vegetated areas, green – forested areas, and brown – areas with non-forest vegetation) are shown following Friedl et al. (2010) and Makarieva et al. (2013a).

We compared temperature differences between land and ocean in six regions in the Northern Hemisphere with pronounced seasonal dynamics of land-ocean temperature contrasts and precipitation (Fig. 2). We additionally considered Sahara and the inner part of the Arabian Peninsula, to enable comparison with some of the driest regions on Earth.

Data for the land cover (Friedl et al., 2010) used in Fig. 2 were downloaded from The Oak Ridge National Laboratory Distributed Active Archive Center at http://daac.ornl.gov/cgi-bin/dsviewer.pl?ds_id=968, which is the International Geosphere-Biosphere Programme (IGBP) Land Cover Data for the 2000–2001 time period. The original land cover data were arranged in 17 classes, which we grouped into four, emphasizing forest versus non-forest vegetation and unvegetated (or sparsely vegetated) regions like deserts and urban areas (see Makarieva et al., 2013a, their Online Resource).

The oceanic and terrestrial parts of the regions were chosen to be of equivalent size and latitude and to exceed in length the characteristic exponential length scale of precipitation decline inland (a few hundred kilometers, see Makarieva et al., 2009). (For the inner part of the Arabian Peninsula, the nearest water body of comparable size is the Mediterranean Sea, which has a different latitude.) We focused on the Northern Hemisphere as it harbors most landmasses that have been experiencing most warming as compared to the oceans (e.g., Rohde and Hausfather, 2020, their Fig. 4). Therefore, the land-ocean temperature contrasts in the Northern Hemisphere have increased in recent decades possibly approaching the threshold that we aim to investigate.

We used NCEP Reanalysis Derived data concerning the long term monthly means (derived from years 1981 to 2010) of air temperature, relative humidity and zonal and meridional wind at the surface and geopotential height and air temperature at 13 pressure levels (from 1000 to 70 hPa), as provided by the NOAA/OAR/ESRL PSD, Boulder, Colorado, USA from their website at https://psl.noaa.gov/data/gridded/data.ncep.reanalysis.derived.html (Kalnay et al., 1996). The data represent $2.5^{\circ }\times 2.5^{\circ }$ global grids.

**Figure 3:**
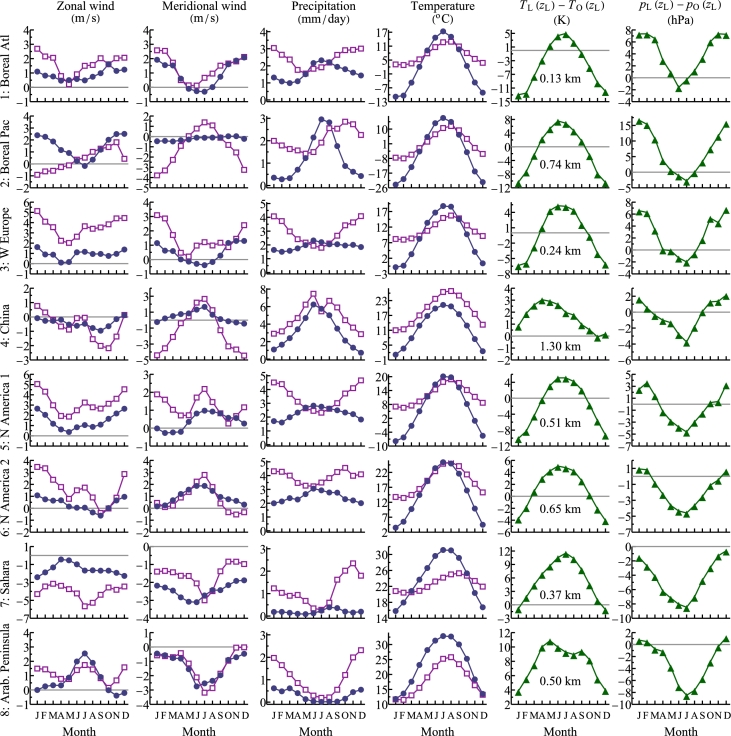
Zonal and meridional wind, mean monthly precipitation and surface temperatures on land (circles) and over the ocean (squares) in the studied regions, the temperature difference between land and ocean at the mean land elevation *z*_L_ (shown in the fifth column) and the mean pressure difference at the same altitude. Note that *T*_L_(*z*_L_)−*T*_O_(*z*_L_) is not equal to the difference in surface temperatures in the fourth column.

Our analysis followed the procedure introduced by Makarieva et al. (2015). For each month, we averaged temperatures and geopotential heights at the 13 pressure levels separately over land and over the ocean and, by interpolation, obtained vertical profiles of mean temperature $T_{L}(z)$ and pressure $p_{L}(z)$ on land and over the ocean, $T_{O}(z)$ and $p_{O}(z)$. From these profiles we calculated the temperature and pressure differences between the air over land and the air over the ocean at the mean height $z_{L}$ of land surface above the sea level. These temperature and pressure differences are shown for each region in the fifth and sixth columns in Fig. 3, respectively.

The minimal pressure difference between land and ocean occurs in June in the Atlantic boreal region and in July in the remaining regions (Fig. 3, sixth column). The maximum of precipitation occurs nearly simultaneously with the minimal pressure difference: in July in the two boreal regions, in June in Western Europe and China, and in August in Sahara (Fig. 3, third column). These rainfall maxima on land are close in time with rainfall minima over the ocean, which indicates ocean-to-land moisture transport (Fig. 3, third column). In contrast, the rainfall maxima on land and over the sea coincide in the Arabian region, where moisture transport is negligible.

The temperature differences between land and ocean are maximum during the summer months (Fig. 3, fifth column) except in China. The China region has the highest elevation ($z_{L}=1.3$ km) among the four regions. Its land surface is always colder than the oceanic surface, but it is warmer than the atmosphere over the ocean at equivalent elevation (Fig. 3, fourth vs fifth column). The meridional wind in China increases during maximum rainfall both over land and over the ocean indicating moisture transport from southern regions rather than from the same latitude.

**Figure 4:**
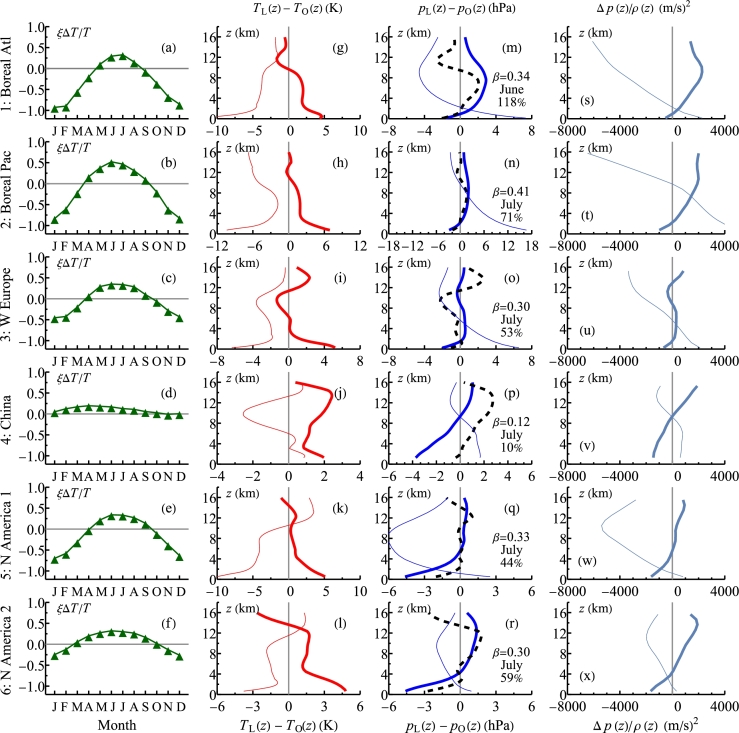
Parameters of Eqs. (14)–(17). First column: seasonality of the temperature change term in Eq. (14), with Δ*T*/*T* = (*T*_L_(*z*_L_)−*T*_O_(*z*_L_))/*T*_O_(*z*_L_) and *ξ* = *L*/(*RT*_O_(*z*_L_)). Second and third columns: vertical profile of the mean temperature differences between air over land and over the ocean during the month with maximum rainfall (thick solid curve) and January (thin curve). In the third column, solution *p*_A_(*β*,*z*)−*p*_D_(*z*) (hPa) of Eqs. (15)–(17) is shown with a dashed curve with the corresponding *β*, month of the minimal pressure difference Δ*p*_obs_ (from Fig. 3, sixth column) and the ratio of theoretical to observed pressure differences Δ*p*(*β*)/Δ*p*_obs_ × 100% indicated in the graph. Fourth column: kinetic energy corresponding to the horizontal pressure differences as dependent on altitude *z* for the month with maximum rainfall (thick curve) and January (thin curve) (see Discussion).

We estimated *β* in Eq. (14) for the month with the minimal pressure difference from the condition that the pressure difference between two hydrostatic air columns with the vertical temperatures profiles $T_{L}(z)$ and $T_{O}(z)$ (Fig. 4, second column) and the pressure difference at $z_{L}$ equal to Δp(β), turns to zero at height $z_{0}$ where the ratio of local $p_{v}(z_{0})$ to surface $p_{v}(z_{L})$ equals *β*:

(15) $$p_{A}(\beta ,z_{0})-p_{D}(z_{0})=0,\;\beta =\frac{p_{v}(z_{0})}{p_{v}(z_{L})},$$

where

(16) $$p_{A}(\beta ,z)\equiv \left[p_{O}(z_{L})+\Delta p(\beta )\right]\exp[-(z-z_{L})h_{L}^{-1}],\;h_{L}\equiv \frac{RT_{L}(z)}{Mg},$$

(17) $$p_{D}(z)\equiv p_{O}(z_{L})\exp[-(z-z_{L})h_{O}^{-1}],\;h_{O}\equiv \frac{RT_{O}(z)}{Mg}.$$

The ratio of water vapor partial pressures at $z_{0}$ and $z_{L}$ in Eq. (15) was calculated from the Clausius-Clapeyron equation (6) assuming that at $z_{0}$ water vapor is saturated and at $z_{L}$ the relative humidity is equal to the mean monthly relative humidity. Equations (15)–(17) represent an algorithm of calculating *δT* for any pair of ocean (donor) and land (acceptor) regions with any type of vegetation cover.

## 5 Results: longitudinal land-ocean temperature contrasts in the Northern Hemisphere

The *β* values obtained by solving Eq. (15) numerically are shown in the third column of Fig. 4 together with the actual pressure difference profiles. These solutions correspond to $z_{0}$ below 3 km and *β* values ranging from 0.30 to 0.41 (except in China where β=0.12 possibly due to its higher elevation, see discussion above). Similar values are obtained for the June and August temperature profiles (data not shown). These findings are consistent with the observation that most kinetic power in the extratropical atmosphere is generated below the 800 hPa level (e.g., Makarieva et al., 2017, Fig. A1c,d).

We emphasize that the existence of the solutions to Eq. (15) and their plausibility is not guaranteed *a priori*. The ratio of CIAD pressure difference Δp(β) to the observed pressure difference $\Delta p_{\mathrm{obs}}$ is shown in Fig. 4, third column. It ranges from 44% to 118% except in China where it is 10% for the reasons discussed above. These new results, i.e., that the theoretical CIAD pressure difference is comparable to the observed pressure difference, support the relevance of the underlying theory.

Using the estimated $\Delta T\equiv T_{L}(z_{L})-T_{O}(z_{L})$ for the month with maximum rainfall in each region, we can find additional temperature difference *δT* that would turn Δ*p* in Eq. (14) to zero and block atmospheric moisture transport to the region:

(18) $$\delta T(\beta )\equiv \frac{\beta }{1-\beta }\frac{T}{\xi }-\Delta T.$$

**Figure 5:**
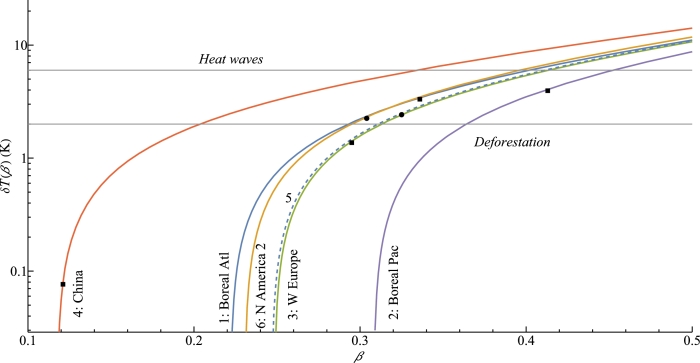
Solutions of Eq. (18) for the six studied regions: *δT* is the additional temperature difference between land and ocean at which the condensation-induced moisture transport is expected to discontinue. Solid squares (for the North American regions – circles) indicate the *β* values from Fig. 4, fourth column. Solution for region 5: N America 1 is shown by the dashed curve to better discern it from region 3. The two horizontal lines indicate a temperature anomaly (6 K) characteristic of recent heatwaves in the Northern Hemisphere (Philip et al., 2021), and the temperature difference (∼2 K) caused by (partial) cutting of native forests (Baker and Spracklen, 2019; Alkama and Cescatti, 2016). The black square for Western Europe located slightly below the “Deforestation” line indicates that at the estimated *β* = 0.3 in July the two degrees of extra warming due to deforestation can potentially prevent regular moisture transport. For the two North American regions a slightly greater warming is required.

These solutions are shown in Table 2 and Fig. 5. The obtained results highlight the following differences between the vegetated (1-6) and practically unvegetated (7-8) regions. First, at time of maximum precipitation, the land-ocean temperature differences in the unvegetated regions are by a few degrees Kelvin higher than in the vegetated regions. Second, despite these higher land-ocean temperature contrasts, maximum precipitation over the unvegetated regions is only about one fourth or fifth of the maximum precipitation over the ocean (sea), while over the vegetated regions it is not less than approximately two thirds of maximum oceanic rainfall (Table 2, 7th column). Furthermore, during the month of maximum precipitation, precipitation over land in the unvegetated regions is approximately one half of what it is over the ocean (sea). Conversely, in the vegetated regions, precipitation over land is larger than, or approximately the same as, over the ocean (Table 2, 8th column). While our first analysis has employed only rough division into vegetated and (largely) unvegetated land, the developed framework can be further applied to a more detailed classification of vegetation cover types.

**Table 2:** The temperature difference surplus to block moisture import, *δT*, and related climatic parameters, in the studied regions.

Region	*β*	*δT*(*β*)	$\Delta T_{\max}$	Δ*T*_max_ + *δT*(*β*)	*P*_max_	$P_{\max}/P_{\max}^{o}$	*P*_max_/*P*^o^
		(K)			(mm/day)		
1: Boreal Atl	0.34	3.4	4.3	7.8	2.3	0.76	1.20
2: Boreal Pac	0.41	4.1	6.9	11.0	3.0	1.00	1.50
3: W Europe	0.30	1.4	5.2	6.6	2.3	0.57	1.20
4: China	0.12	0.1	2.0	2.0	6.2	0.84	0.84
5: N America 1	0.33	2.5	5.1	7.6	2.8	0.61	1.20
6: N America 2	0.30	2.3	4.8	7.1	3.1	0.67	0.95
7: Sahara			12.0		0.4	0.17	0.65
8: Arabian Pen.			9.3		0.6	0.27	0.31

Here *β* and δT(β) are calculated from Eqs. (15) and (18), respectively; $\Delta T_{\max}$ is the maximum monthly temperature difference between land and ocean at mean land height $z_{L}$ (see Fig. 3, fifth column), $P_{\max}$ and $P_{\max}^{o}$ are the maximum monthly precipitation rates over land and ocean, respectively (see Fig. 3, third column); $P^{o}$ is the monthly precipitation over the ocean when the monthly precipitation over land is maximum.

One can see that the long-term temperature contrasts during the warm season in the Northern Hemisphere are close to the threshold where the condensation-induced moisture transport ceases. These contrasts can be driven beyond the threshold by temperature anomalies characteristic of recent heatwaves (Fig. 5). At the observed *β* values a few degrees extra warming of land can cause the condensation-induced moisture transport to stop (Fig. 5). For example, for Western Europe a warming of 1.4 K would be sufficient (Table 2, third column). Extra warming on land is associated with heatwaves and blocking anticyclones (e.g., Chan et al., 2019). During heatwaves the temperature anomaly may reach six degrees Celsius (e.g., Philip et al., 2021). In this situation, the surface cooling provided by transpiring vegetation can be crucial to avoid a tipping point where the climate switches to arid. Natural, undisturbed forests provide the strongest buffer against surface warming (Alkama and Cescatti, 2016; Baker and Spracklen, 2019). Disturbance of the forest cover by deforestation increases summer temperatures by up to two degrees (Baker and Spracklen, 2019; Alkama and Cescatti, 2016).

Schematically, removing forests can initiate a dangerous feedback: as the horizontal temperature contrasts grow, moisture transport declines which further increases the temperature surplus. If the descending air in a blocking anticyclone has created a critical temperature surplus, in the absence of vegetation nothing can disrupt the resulting circulation. Conversely, a water-sufficient sustainable forest can forcefully cool the area through transpiration and thus reduce the excess heat. A large-scale example of this process in action is provided by the Amazon rainforest that promotes the onset of the wet season by enhancing transpiration during the dry season (Wright et al., 2017). As forest transpiration increases, the land temperature declines (e.g., Wright et al., 2017, their Fig. 2C).

Droughts and other disruptions of the water cycle compromise the forest capacity to contribute to climate stabilization (Sheil et al., 2019). There is a long-term legacy from past land-use practices that determines ecosystem's response to current climate (Aleinikov, 2019; Buras et al., 2020). It is therefore crucial to differentiate ecosystems capable of self-recovery from those on the degradation trajectory. Once disrupted, given the large time scale of forest successional recovery towards the natural state, the moisture-regulating functions of intact forests cannot be rapidly restored. Given the complex vegetation properties and the processes that prevent abrupt landscape transitions from wetness to aridity (Fig. 1), a random replacement of intact forests by artificial plantations is not likely to recover the water cycle stability. This may explain mixed success of large-scale afforestation/rewetting efforts in China (Jiang and Liang, 2013; Ahrends et al., 2017; Zinda and Zhang, 2019; Zhao et al., 2021).

Our analysis could have revealed that the values of *β* and Δ*T* in Eq. (18), as estimated from observations, are, respectively, too large and too small. Then the observed temperature anomalies $\delta T_{\mathrm{obs}}$ (due to deforestation or heatwaves) would be much smaller than δT(β) and have a negligible impact on the parameters of the condensation-induced moisture transport. Instead, we found that these values are such that $\delta T_{\mathrm{obs}}∼\delta T(\beta )$. This means that a disruption of the atmospheric water transport by extra warming is plausible and could be responsible for the increasing frequency of extreme events. It is a new result and a prediction to be evaluated.

## 6 Discussion

We have shown that within the framework of condensation-induced atmospheric dynamics, wind power is reduced when horizontal air motion occurs in the direction of higher temperatures. Addition of water vapor by evaporation in the horizontal flow partially compensates for the pressure drop by condensation in the ascending air. This reduces the release of potential energy to power winds. The generation of wind power (the scalar product of wind velocity and pressure gradient) is what sustains wind despite energy being lost to friction. Once the temperature gradient becomes sufficiently large, the condensation-induced wind power tends to zero and the related air flow ceases.

Consideration of possible changes in the ocean-to-land circulation (Fig. 1) is conventionally made in terms of the temperature effects (the “breeze-like” circulation, see Hill, 2019). In a hydrostatic atmosphere, other things being equal, a higher surface temperature creates a pressure surplus in the upper atmosphere that pushes the air away from the warmer air column. This air outflow reduces total air mass and produces a pressure shortage at the surface that causes the low-level air convergence towards the warmer area. However, in this consideration the magnitude of the resulting air inflow and whether the warming is efficient enough to explain the observed convergence, cannot be unambiguously quantified (e.g., Lindzen and Nigam, 1987). This conventional qualitative picture does not rule out other mechanisms.

Condensation-induced atmospheric dynamics provides a distinct mechanism to reduce surface pressure and to power the low-level moisture convergence, but it does not specify a mechanism for the upper-level outflow. Figure 4 (fourth column) shows that the kinetic energies required to move against the upper tropospheric temperature-related pressure differences are in the order of $10^{3}\;{\text{m}}^{2}{\text{/s}}^{2}$. Such energies are present in tropical cyclones but not in large-scale transcontinental air circulation. For the CIAD to be realized, some mechanism for the outflow should be present: either the differential warming, or latent heat release, or, as in hurricanes, the cyclostrophic imbalance. This results in condensation being often concentrated in the warmer regions (unless, like in Ferrel cells, there is an external dynamical driver to push the upper air against the pressure surplus in the upper atmosphere (Makarieva et al., 2017)).

This coupling of condensation-induced atmospheric dynamics with conventional mechanisms (like a higher moisture inflow towards a warmer land surface) masks its presence in the conventional qualitative picture and could be the reason for CIAD having been neglected. That it produces realistic quantitative estimates of wind power is an indication that without CIAD the same outflow mechanisms would have generated weaker circulations.

The differential warming of land versus the ocean and the preferential release of latern heat either over land or over the ocean have different implications for the resulting circulations. It has been recognized, since the works of Charney (1975), that a warm land surface does not necessarily initiate a moisture inflow if the land is also dry: the surface warming will be negated by cooling of the adiabatically ascending dry air (Fig. 1a). However, if the land is both warm and moist, the conditions for ocean-to-land moisture inflow are conventionally considered favorable. We show that this may not always be the case. Too high land-ocean temperature contrasts can inhibit, and block, moisture inflows and the ascending motion of moist air.

This can be a mechanism contributing to the formation of blocking anticyclones and heatwaves, for which, as commonly recognized, there is no comprehensive dynamic theory or understanding (Woollings et al., 2018; Miralles et al., 2019). One of the conceptual questions is the following: if the air rises where it is warm, why does it descend where it is the warmest, i.e., during heatwaves associated with blocking anticyclones? Here the above-described difference between CIAD and temperature-driven circulation provides suggestions.

For the CIAD circulation to work, there must be a pressure drop. This happens when the moist air rises and vapor condenses. As the moist air moves horizontally towards the area of ascent, if the surface is moist and its temperature increases along the streamline, the air will acquire water vapor. If this temperature rise is too high, the amount of acquired water vapor due to evaporation can exceed the amount lost due to condensation. The net pressure difference will be zero, and the CIAD circulation will stop or reverse. (Indeed, heatwaves are accompanied by a spike in evaporation, see, e.g., Sitnov et al., 2014; Miralles et al., 2019, Fig. 2). The prevalence of CIAD mechanisms over the temperature-driven motions will then account for the persistence of the descending air motion in the warmest area.

It is critically important to continue theoretical investigations of moist atmospheric dynamics and the role of vegetation, considering jointly the biotic pump mechanism and the temperature-driven effects. Previously, we indicated that a major role of vegetation in the atmospheric moisture transport is to keep the atmosphere moist via transpiration (Makarieva and Gorshkov, 2007; Makarieva et al., 2014b). Here we highlighted an additional role: to buffer the land-ocean temperature contrasts. Recognizing these physically distinct effects of temperature on air circulation can help us better understand and project the diverse impacts of land cover change on the local and regional water cycle (Lawrence and Vandecar, 2015; te Wierik et al., 2021; Caballero et al., 2022). The effects of the vegetation cover change on the regional water cycle can be exacerbated by atmospheric teleconnections (e.g., the Rodwell-Hoskins mechanism, Rodwell and Hoskins, 1996).

In a broader context, the current international focus on mitigating carbon emissions raises the importance of renewable energy sources and causes an increased pressure on global forest ecosystems (Jonsson and Rinaldi, 2017; Lauri et al., 2017). Deforestation leads to the emission of dioxide which contributes to warming the planet but in the boreal region is considered to cool the planet via an increase in albedo, the overall effect is judged to be small despite the uncertainties (Jia et al., 2019). This narrative has allowed the on-going extirpation of native boreal forests to proceed with little international concern.

Meanwhile, the role of forests in transcontinental moisture transport and in controlling regional temperature regime, has become much clearer (e.g., Nobre et al., 2009; van der Ent et al., 2010; Pielke Sr et al., 2011; Alkama and Cescatti, 2016; Mahmood et al., 2016; Leite-Filho et al., 2021; Meier et al., 2021). Russia, for example, is home to some of the world's most extensive natural forest (Potapov et al., 2008). The pristine forest ecosystems are characterized by resilience to perturbations like fires, windfall or pests (Sukachev, 1975; Gromtsev, 2002; Rich et al., 2007; Shorohova et al., 2008; Debkov et al., 2019). They also stabilize regional and global climates (Gorshkov, 1995; Funk et al., 2019; Makarieva et al., 2020).

Recent research has highlighted how forests buffer downwind regions against fluctuations in precipitation (O'Connor et al., 2021). Conversely, loss of native forest cover should result in continent-scale destabilization of the water cycle and temperature regime. Indeed, simultaneously with pristine forests being lost in Russia (Potapov et al., 2017), the Eurasian continent is drying and increasingly suffering violent winds, floods and droughts (Gu et al., 2019; Krause et al., 2020; Cornwall, 2021). A strategy to mitigate climate change and stabilize the continental water cycle must include a focused research-policy program aimed at protecting natural forests (in Russia, Canada and beyond). As moisture transport ignores political borders making downwind countries highly dependent on upwind vegetation cover (van der Ent et al., 2010), forest conservation and restoration policies in one country (e.g., China) will not be successful if they are accompanied by increased pressure on intact ecosystems in another (e.g., Russia).

While the appreciation of the importance of natural ecosystems is now on the rise (EASAC, 2017; Jonsson et al., 2020; Sabatini et al., 2020), the understanding, and corresponding research, of their active participation in the many aspects of climate stabilization, as well as of the potential of *proforestation* (Moomaw et al., 2019) for climate change mitigation, remain inadequate. Large-scale drought-mitigation measures can only be successful within a broader strategic framework that recognizes the role of forest cover, and pristine forests in particular, in the water cycle and atmospheric dynamics. Elaborating such a framework requires a major interdisciplinary effort.

## 7 Conclusion

We have shown that the continuity equation yields an estimate on wind from a known condensation rate. Minor changes in condensation rate result in marked changes in wind power (Table 1). These results are pertinent to predicting regional changes in the terrestrial water cycle, especially where models disagree even on the sign of changes (e.g., Hill, 2019). Recognizing this sensitivity can improve model parameterizations.

We further derived a theoretical equation, Eq. (14), which describes how the CIAD-induced pressure difference Δp(β) depends on the temperature difference Δ*T* between the donor (ocean) and acceptor (land) regions and on the degree of condensation *β*. Here, *β* is not a free parameter but is determined by the properties of the circulation. It is defined as the relative amount of moisture that has condensed as the moist air reaches height $z_{0}$ where the pressure difference between the donor and acceptor regions changes sign (and thus, above $z_{0}$, there is an air outflow to, rather than inflow from, the ocean).

Therefore, with Δ*T* set by observations, this equation may or may not have realistic solutions. We found that such solutions exist in summer (when precipitation reaches a maximum in the studied regions): the estimated theoretical values of Δp(β) are comparable to the observed pressure differences $\Delta p_{\mathrm{obs}}$ between land and ocean, and so is $z_{0}$ (Fig. 4, third column), indicating that our estimates, and the underlying mechanisms, are realistic.

The case of China, where Δp(β) is relatively small when compared to $\Delta p_{\mathrm{obs}}$, indicates a greater role of the meridional moisture transport from a (relatively) warmer ocean to a colder land. Such circulation requires further investigation.

Furthermore, our theoretical framework indicates the existence of a critical temperature difference ΔT(β), Eq. (18), when Δp(β) becomes zero and the condensation-induced circulation stops. We estimated these critical ΔT(β) values and found that they are only moderately larger than the observed temperature differences in the studied regions.

Ominously, an additional regional surface warming of 1–2 K following loss of vegetation can disrupt the atmospheric moisture transport in the regions we studied. Ecosystem resilience and transpiration are crucial to stabilising and maintaining the regional water cycle. The wellbeing of much of the World's people is threatened by destruction of forest and other natural tree cover.

## Declarations

### Author contribution statement

A.M. Makarieva; A.V. Nefiodov: Analyzed and interpreted the data; Wrote the paper.

A.M. Makarieva; A.V. Nefiodov; A.D. Nobre; D. Sheil; P. Nobre; J. Pokorný; P. Hesslerová; B.-L. Li: Analyzed and interpreted the data.

### Funding statement

This work was funded by the 10.13039/501100002347Federal Ministry of Education and Research (BMBF) and the Free State of Bavaria under the Excellence Strategy of the Federal Government and the Länder, and the 10.13039/501100015072Technical University of Munich's Institute for Advanced Study.

### Data availability statement

Data included in article/supp. material/referenced in article.

### Declaration of interests statement

The authors declare no conflict of interest.

### Additional information

No additional information is available for this paper.

## Appendix A Deriving Eqs. (9) and (12)

For the convenience of our readers here we repeat the derivations of Makarieva et al. (2014a). In the stationary case, the continuity equations for the water vapor and the dry air constituents have the form

(A.1a) $$\nabla \cdot (\text{v}N_{v})\equiv N_{v}(\nabla \cdot \text{v})+(\text{v}\cdot \nabla )N_{v}=S,$$

(A.1b) $$\nabla \cdot (\text{v}N_{d})\equiv N_{d}(\nabla \cdot \text{v})+(\text{v}\cdot \nabla )N_{d}=0,$$

where $N_{v}$, $N_{d}$ and $N=N_{v}+N_{d}$ are the molar densities of water vapor, dry air constituents and moist air as a whole, respectively. Air velocity v=u+w is equal to the sum of the horizontal **u** and vertical **w** velocity components. The quantity S (${\text{mol/m}}^{3}\text{/s}$) represents the volume-specific rate at which molar density $N_{v}$ of water vapor is changed by phase transitions. By multiplying (A.1b) by $\gamma_{d}\equiv N_{v}/N_{d}$ and excluding $N_{v}(\nabla \cdot \text{v})$ from (A.1a), we obtain

(A.2) $$(\text{v}\cdot \nabla )N_{v}-\gamma_{d}(\text{v}\cdot \nabla )N_{d}=S.$$

Using the ideal gas equation of state

(A.3) $$p=NRT,\;p_{v}=N_{v}RT,\;p_{d}=N_{d}RT,$$

one can replace molar densities $N_{i}$ in (A.2) with partial pressures $p_{i}$ (i=v,d) and the rate of phase transitions S with the power of phase transitions σ≡SRT (${\text{W/m}}^{3}$):

(A.4) $$(\text{v}\cdot \nabla )p_{v}-\gamma_{d}(\text{v}\cdot \nabla )p_{d}=\sigma .$$

Owing to the universality of the gas constant *R* the contribution due to temperature gradient ∇*T* cancels.

Substituting (11) into (A.4) and taking into account the identity

(A.5) $$\nabla p_{v}-\gamma_{d}\nabla p_{d}\equiv (1+\gamma_{d})(\nabla p_{v}-\gamma \nabla p),$$

where $\gamma \equiv p_{v}/p\equiv \gamma_{d}/(1+\gamma_{d})$, we obtain the following relation for (A.4):

$$(\text{w}+\text{u})\cdot (\nabla p_{v}-\gamma \nabla p)=\frac{1}{1+\gamma_{d}}(\text{u}\cdot \nabla )p.$$

By transferring γ(u⋅∇)p to the right-hand side of the last relation and taking into account relation between *γ* and $\gamma_{d}$, we obtain:

(A.6) $$p(\text{w}\cdot \nabla )\gamma +(\text{u}\cdot \nabla )p_{v}=(\text{u}\cdot \nabla )p,\;p\nabla \gamma \equiv \nabla p_{v}-\gamma \nabla p,$$

which coincides with Eq. (12).

## Footnotes

1 While turbulent diffusion is not explictly accounted for in the continuity equation, it is implicitly present in the condition $\partial p_{v}/\partial x=0$, i.e., turbulent diffusion is what ensures constant relative humidity on an isothermal plane.

2 This term represents an additional work by gravity associated with the fact that it is the lighter gas (water vapor) that is compressed in the vertical relative to equilibrium. With $h_{c}≃2$ km, $h_{d}≃h≃10$ km and $M_{v}/M_{d}≃0.6$ this term increases the absolute magnitude of the wind power by approximately 10%.
